# Extensively drug-resistant tuberculosis in Togo: first reported cases and implications for tuberculosis control

**DOI:** 10.1016/j.ijregi.2025.100825

**Published:** 2025-12-17

**Authors:** Maïssala Zoutené, Akouvi Mawussé Edjodjinam Ako, Koffi Atsu Aziagbe, Narcisse Viani Gateu Tadjom, Tété Amento Stéphane Adambounou, Komi Séraphin Adjoh

**Affiliations:** 1 Department of Pulmonology, Sylvanus Olympio University Hospital, Lomé, Togo; 2 Faculty of Health Sciences, University of Lomé, Lomé, Togo

**Keywords:** Tuberculosis, Drug resistance, Togo

## Abstract

**Objectives:**

The increasing burden of extensively drug-resistant tuberculosis (XDR-TB) undermines global TB control efforts.

**Methods:**

This was a case series study conducted from January 1, 2007, to December 31, 2024, in the Department of Pulmonology at Sylvanus Olympio University Teaching Hospital.

**Results:**

We report a series of three cases. Case 1: A 30-year-old man with a history of contact with an XDR-TB case was treated with a 20-month regimen. Culture conversion was achieved at the 3^rd^ month of treatment. A complication in the form of pyopneumothorax occurred during the 6^th^ month of therapy. Case 2: A 51-year-old patient with no significant medical history was diagnosed with XDR-TB after 4 months of treatment for multidrug-resistant TB (MDR-TB). Conversion of follow-up cultures was achieved 2 months after modification of the treatment regimen. Case 3: A 62-year-old woman living with human immunodeficiency virus (HIV), previously treated for MDR-TB, developed XDR-TB during the course of treatment. The patient died on the 29th day of XDR-TB treatment.

**Conclusions:**

XDR-TB is a curable disease. Early and accurate diagnosis allows for better selection of the most appropriate treatment strategy.

## Highlights

• The increase in cases of extensively drug-resistant tuberculosis in resource-limited countries is undermining World Health Organization efforts.
• A series of three cases, including two patients not infected with HIV.
• The varying length of time taken to diagnose cases in resource-limited countries.
• The complexity of treatment and the toxicity of aminoglycosides.

## Introduction

Extensively drug-resistant tuberculosis (XDR-TB) was previously defined as multidrug-resistant TB (MDR-TB) with additional resistance to fluoroquinolones and at least one of the three injectable second-line anti-TB drugs (amikacin, capreomycin, or kanamycin) [1]. Since 2022, the World Health Organization (WHO) has redefined XDR-TB as MDR-TB with additional resistance to fluoroquinolones and to at least one Group A drug (linezolid or bedaquiline) [2].

In 2023, an estimated 10.8 million people were infected with *Mycobacterium tuberculosis*, with a proportion of MDR/rifampicin-resistant TB (MDR/RR-TB) of 3.2% among new cases and 16.0% among previously treated cases. A total of 28,982 individuals were affected by pre-extensively drug-resistant TB (pre-XDR-TB) or XDR-TB [3]. TB caused 1.25 million deaths worldwide in 2023 [3].

Unlike drug-susceptible TB, the treatment of drug-resistant TB is prolonged and relies on more complex regimens involving more toxic drugs, often associated with severe adverse effects. While the management of MDR-TB has shifted toward shorter, fully oral treatment regimens, these regimens have not been evaluated in pre-XDR-TB and XDR-TB. These forms of TB have limited therapeutic options, lack standardized treatment regimens, and are associated with low treatment success rates [4,5].

In Togo, a resource-limited country, the prevalence of MDR/RR-TB is estimated at 0.6% among new cases and 3.0% among previously treated cases [6]. This study aims to describe the clinical characteristics and outcomes of the first reported cases of XDR-TB in Togo.

## Methods

We conducted a case series study from January 1, 2007, to December 31, 2024, in the Department of Pulmonology at Sylvanus Olympio University Teaching Hospital, the national referral center for the management of drug-resistant TB in Togo. Data were collected retrospectively. Medical records were reviewed to obtain demographic and clinical history data (age, sex, comorbidities), as well as microbiological and radiological findings. Treatment effectiveness and tolerability were assessed based on clinical and microbiological data available in the medical records. The review of medical records was initiated after obtaining authorization from the Head of the Department of Pulmonology.

## Results

**Figure 1:**
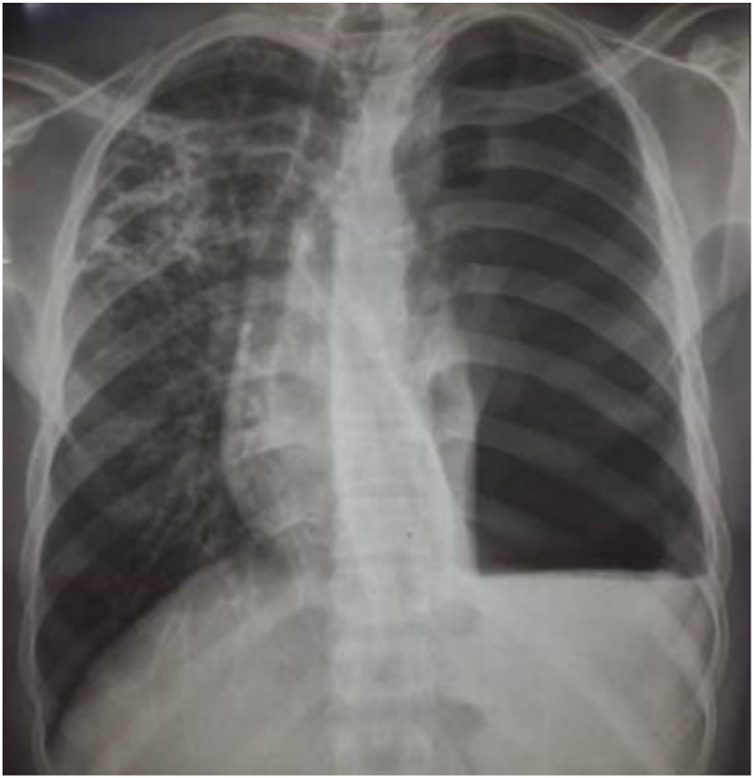
Chest X-ray showing a left hydropneumothorax secondary to extensively drug-resistant tuberculosis in case 1.

**Case 1 (diagnosed on May 15, 2019):** This was a 30-year-old man, weighing 67 kg, living in Lomé, immunocompetent with respect to HIV, and known to have chronic hepatitis B, with a history of contact with an XDR-TB case. The illness began 2 months prior to admission with cough, dyspnea, chest pain, fever, and weight loss. Physical examination on admission revealed bilateral pulmonary consolidation syndrome. Chest radiography showed bilateral alveolar infiltrates with cavitary lesions in the left lung. Sputum smear microscopy for acid-fast bacilli (AFB) was positive (3+) on direct examination, and the Xpert MTB/RIF assay detected *M. tuberculosis* with rifampicin resistance. A second-line anti-TB regimen was initiated, consisting of 4 months of amikacin (1 g/day), moxifloxacin 400 mg (1 tablet/day), prothionamide 250 mg (2 tablets/day), isoniazid 300 mg (1.5 tablets/day), clofazimine 100 mg (1 tablet/day), ethambutol 400 mg (2 tablets/day), and pyrazinamide 400 mg (3 tablets/day), followed by 5 months of moxifloxacin, clofazimine, ethambutol, and pyrazinamide. Line probe assay (LPA) testing revealed resistance to isoniazid, fluoroquinolones, and aminoglycosides, confirming the diagnosis of XDR-TB. The patient was subsequently switched to a 20-month regimen, including a 6-month intensive phase with bedaquiline 100 mg (4 tablets daily for the first 2 weeks, followed by 2 tablets three times weekly, continued for an additional 6 months during the continuation phase), delamanid 50 mg (2 tablets twice daily), isoniazid 300 mg (2 tablets daily), linezolid 600 mg (1 tablet daily), pyrazinamide 400 mg (3 tablets daily), and clofazimine 100 mg (1 tablet daily). The continuation phase consisted of 14 months of linezolid, clofazimine, and pyrazinamide. At the 6^th^ month of treatment, the patient developed sudden onset left-sided chest pain. Chest radiography revealed a compressive left hydropneumothorax (Figure 1). Pleural exploration yielded purulent fluid, and pleural drainage was performed. Follow-up sputum smears were positive (1+) during the 1^st^ month of treatment and showed traces in the 2^nd^ month, with conversion to negative at the 3^rd^ month. Cultures remained positive during the first two follow-up assessments, then converted to negative at the 3^rd^ month and remained negative until the end of treatment. The patient developed lower limb paresthesias, which resolved favorably with pyridoxine supplementation.

**Figure 2:**
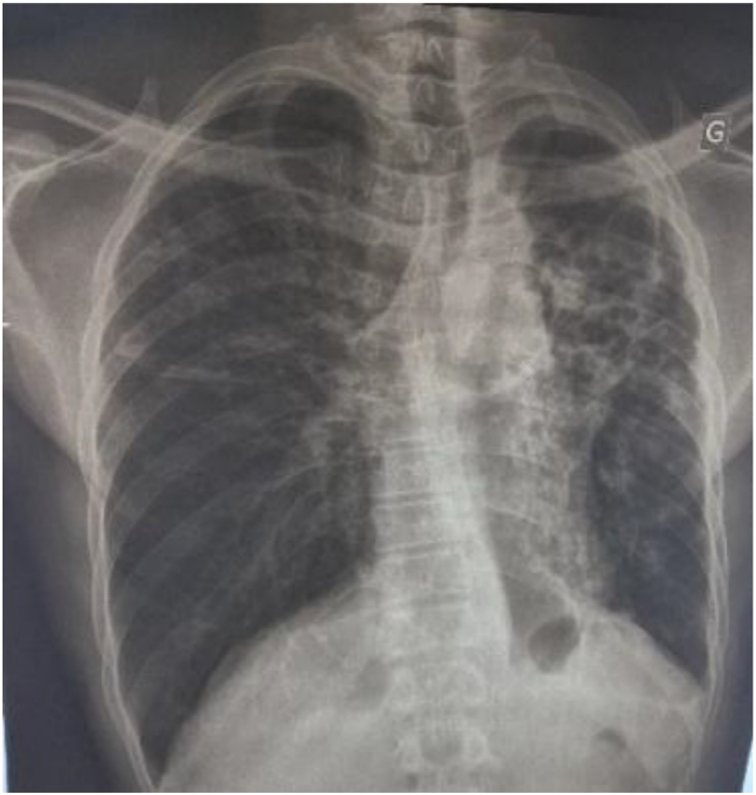
Chest X-ray showing left-sided cavitary and alveolar lesions at the time of diagnosis in case 2.

**Case 2 (diagnosed on March 4, 2019):** This was a 51-year-old man with a negative HIV serology and no history of TB contact, referred from Aného Hospital for drug-resistant TB detected by the Xpert MTB/RIF assay. The patient weighed 50 kg at admission. Physical examination revealed left-sided crackles. Chest radiography showed left-sided cavitary and alveolar lesions with signs of lung retraction (Figure 2). Second-line anti-TB treatment was initiated, consisting of 4 months of amikacin (1 g/day), moxifloxacin 400 mg (1 tablet/day), prothionamide 250 mg (3 tablets/day), isoniazid 300 mg (2 tablets/day), clofazimine 100 mg (1 tablet/day), ethambutol 400 mg (3 tablets/day), and pyrazinamide 400 mg (4 tablets/day), followed by 5 months of moxifloxacin, clofazimine, ethambutol, and pyrazinamide. Follow-up cultures during the first 4 months remained positive, showing resistance to rifampicin and isoniazid. LPA performed on the 4^th^-month specimen demonstrated resistance to fluoroquinolones, amikacin, and kanamycin, confirming the diagnosis of XDR-TB. A 20-month regimen was subsequently initiated, including a 6-month intensive phase with bedaquiline 100 mg (4 tablets daily for the first two weeks, followed by 2 tablets 3 times weekly, continued for an additional 6 months during the continuation phase), delamanid 50 mg (2 tablets twice daily), isoniazid 300 mg (1.5 tablets/day), linezolid 600 mg (1 tablet/day), pyrazinamide 400 mg (2.5 tablets/day), and clofazimine 100 mg (1 tablet/day). The continuation phase consisted of 14 months of linezolid, clofazimine, and pyrazinamide. Follow-up culture was positive at the 1^st^ month, with conversion to negative at the 2^nd^ month, and remained negative until the 20^th^ month of treatment. During the course of treatment, the patient developed bilateral hearing loss.

**Figure 3:**
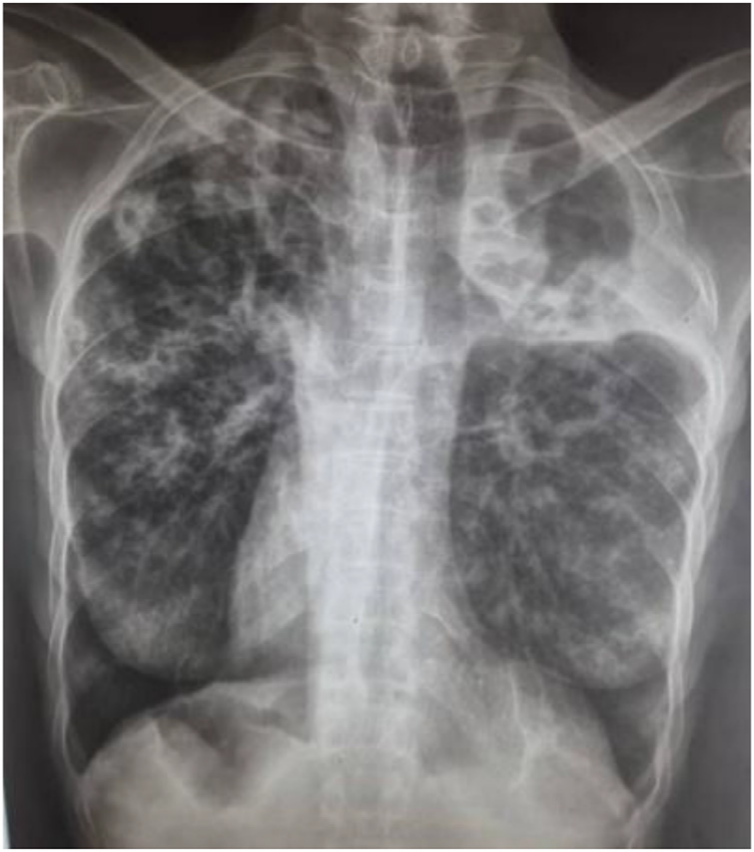
Chest X-ray showing bilateral alveolo-interstitial and cavitary lesions at the time of diagnosis in case 3.

**Case 3 (diagnosed on April 6, 2019):** This was a 62-year-old woman residing in Lomé, living with HIV, and receiving antiretroviral therapy (tenofovir/lamivudine/efavirenz), with a past medical history of intercostal herpes zoster. Her weight at admission was 31 kg. She was being followed for MDR-TB and was receiving a second-line regimen consisting of 4 months of amikacin (1 g/day), moxifloxacin 400 mg (1 tablet/day), prothionamide 250 mg (2 tablets/day), isoniazid 300 mg (1.5 tablets/day), clofazimine 100 mg (1 tablet/day), ethambutol 400 mg (2 tablets/day), and pyrazinamide 400 mg (3 tablets/day), followed by 5 months of moxifloxacin, clofazimine, ethambutol, and pyrazinamide. On admission, physical examination revealed bilateral pulmonary consolidation. Chest radiography showed bilateral alveolo-interstitial and cavitary lesions (Figure 3). Cultures from the first 4 months remained positive, demonstrating resistance to rifampicin and isoniazid. The patient developed bilateral hearing loss during treatment. LPA performed on the 4^th^-month specimen revealed resistance to fluoroquinolones and kanamycin, confirming the diagnosis of XDR-TB. The patient was subsequently switched to a 20-month regimen including bedaquiline 100 mg (4 tablets daily for the first 2 weeks, followed by 2 tablets three times weekly), delamanid 50 mg (2 tablets twice daily), isoniazid 300 mg (1 tablet/day), linezolid 600 mg (1 tablet/day), pyrazinamide 400 mg (2 tablets/day), and clofazimine 100 mg (1 tablet/day). Death occurred on the 29th day of this regimen, in the context of acute dyspnea with oxygen desaturation.

## Discussion

We report three cases of XDR-TB, including one case in a 62-year-old woman living with HIV (Table 1).

**Table 1:** Characteristics of the patients.

	Case 1	Case 2	Case 3
Sex	Male	Male	Female
Age (years)	30	51	62
HIV serology	Negative	Negative	Positive
Diagnostic delay (months)	5	7	6
Outcome	Cured	Cured	Deceased

Limited access to molecular diagnostic tests and the long turnaround time of LPA testing represent the main limitations of our study. WHO-recommended short treatment regimens are not applicable to XDR-TB. The authorization of the short MDR-TB regimen in cases of rifampicin resistance detected by the Xpert MTB/RIF assay may lead to inappropriate treatment, potentially resulting in unfavorable outcomes. Our findings confirm the presence of XDR-TB in Togo, following cases previously reported in other sub-Saharan African countries [7,8]. Among our patients, two were men. Similarly, the two patients reported by Saleri et al. [8] in Burkina Faso were male, whereas Diarra et al. [9] reported two cases in Mali, both in women. A history of contact with an XDR-TB case in one of our patients is consistent with the findings of El Achkar et al. [10], who reported a history of prior treatment in two of their three patients. In contrast, Gandhi et al. [11] found no previous history of treatment in 55.0% of their patients. Previous exposure to second-line anti-TB treatment and a family history of TB are recognized risk factors for XDR-TB [12]. One of our three patients was living with HIV. Similarly, one of the two patients reported by Diarra et al. [9] in Mali was HIV-positive, whereas the patient described by Bakayoko et al. [7] in Côte d’Ivoire was HIV-negative. However, high prevalences of HIV infection among XDR-TB patients have been reported in South Africa [13,14]. Clinical outcomes were favorable in two of our patients, with culture conversion achieved as early as the 1^st^ month of follow-up in one patient and by the 3^rd^ month in the other. Keshavjee et al. [15] reported favorable outcomes in 48.0% of cases. Our HIV-infected patient died on the 29th day of treatment, consistent with the findings of Gandhi et al. [13], who reported a median survival of 28.4 days after sputum collection. In two of our patients, the diagnosis of XDR-TB was established 4 months after initiation of second-line treatment. At that time, limited availability and performance of rapid molecular tests meant that they were not systematically performed. WHO recommends drug susceptibility testing prior to initiating an appropriate treatment regimen [5]. Limited access to rapid molecular diagnostic tests in resource-limited settings such as Togo remains a major challenge to implementing these recommendations. We observed two cases of bilateral hearing loss (Cases 2 and 3). Keshavjee et al. [15] reported ototoxicity in 10.0% of patients. The hearing loss observed in our patients is likely attributable to prior exposure to anti-TB regimens containing aminoglycosides.

## Conclusion

XDR-TB is treatable and potentially curable. Early and accurate diagnosis allows for optimal selection of the most appropriate treatment regimen. Strengthening diagnostic capacity, ensuring rapid access to new drugs such as bedaquiline, implementing systematic contact screening, and maintaining active surveillance are critical factors that should be considered by national TB control programs, including that of Togo.

## Declaration of competing interest

The authors have no competing interests to declare.
